# A New Neotropical Species of *Gnathostoma* (Nematoda: Gnathostomatidae) from the Northern Four-Eyed Opossum *Philander vossi* (Marsupialia)

**DOI:** 10.1007/s11686-025-01155-3

**Published:** 2025-11-14

**Authors:** M. Á. Mosqueda-Cabrera, E. Sánchez-Miranda, L. D. Castillo-Loeza, G. Torres-Carrera, L. García-Prieto

**Affiliations:** 1 Departamento El Hombre y su Ambiente, División de Ciencias Biológicas y de la Salud, Universidad Autónoma Metropolitana-Xochimilco, Calzada del Hueso # 1100, Col. Villa Quietud, Coyoacán, 04960 Mexico City, Mexico; 2 Posgrado en Ciencias Biológicas, Instituto de Biología, Universidad Nacional Autónoma de México, 04510 Mexico City, Mexico; 3 Colección Nacional de Helmintos, Departamento de Zoología, Instituto de Biología, UNAM, Mexico City, Mexico

**Keywords:** Neotropical, *Gnathostoma mexicanum* n. sp., Mammals, Mexico

## Abstract

**Purpose:**

This work provides a detailed morphological description of a previously identified but unnamed lineage inside *Gnathostoma* spp. from Mexico.

**Methods:**

Specimens of the four-eyed opossum *Philader vossi* were collected in Tlacotalpan, Veracruz, Mexico. Specimens were examined morphologically using both light microscopy and scanning electron microscopy. Additionally, we generate DNA sequences for the following loci: mitochondrial *cox*1 and nuclear 5.8S rRNA and ITS-2 and 28S rDNA.

**Results:**

The new species, *Gnathostoma mexicanum* n. sp., differs from *G. turgidum* Stossich, 1902, the common gnathostomid species infecting *Didelphis* spp. from the Americas, in its smaller body size, fewer teeth on the cuticular spines at anterior half of body, as well as site of infection (pyloric region vs. stomach layers). Host specificity further distinguishes the two species. Additionally, molecular data show that the new species clearly diverges from its congeners.

**Conclusions:**

This work represents the fourth *Gnathostoma* species described for Mexican mammals and the eight recorded in the Americas.

## Introduction

Nematodes belonging to the genus *Gnathostoma* Owen, 1837 have been extensively investigated, in part because some species can cause zoonotic disease when third-stage larvae are ingested through undercooked intermediate hosts such as fish or frogs [1, 2]. Naturally, *Gnathostoma* species infect mammals, primarily carnivores or marsupials, though they have also been recorded in pigs [3, 4].

Currently, there are 13 valid species of *Gnathostoma*, seven of which ranging in the American continent [5]: *Gnathostoma sociale* Leidy, 1858 from *Mustela vison* of USA [6]; *Gnathostoma procyonis* Chandler, 1942 infecting *Procyon lotor* of south USA [7]; *Gnathostoma lamothei* Bertoni-Ruiz et al. [3] (= *Gnathostoma* sp. I sensu [8], and = *G. procyonis *sensu [9]) in *P. lotor hernandezii* of Mexico); *Gnathostoma binucleatum* Almeyda-Artigas, 1991 in *Leopardus pardalis* and *Felis catus* from Mexico and Ecuador [8–11]; *Gnathostoma americanum* Travassos, 1925, a parasite of *Leopardus tigrinus* from Brazil; and *Gnathostoma turgidum* Stossich, 1902 (= *G. didelphis* Chandler, 1932), [5, 8, 12]. All of these species parasitize the stomach of their host, except for *Gnathostoma miyazakii* Anderson, 1964, which was found in the kidney tissue of *Lontra canadensis* in Canada and the USA [13, 14].

Following a detailed morphological and molecular study of *Gnathostoma* specimens collected in Mexico in the Northern four-eyed opossum, *Philander vossi*, we identified an undescribed species of this genus. This work aims to describe this new species, differentiating it from the seven known American *Gnathostoma* species, based on morphological and molecular evidence.

## Materials and Methods

### Specimen Collection

Eight specimens of *P. vossi* collected between April 2022 and March 2023 under collection permit SCPA/DGVS/03184/22 issued by the Secretaría de Medio Ambiente y Recursos Naturales, Mexico, were necropsied for helminths. Briefly, Tomahawk traps baited with a mixture of tuna and canned bananas were placed near freshwater bodies around Tlacotalpan city (18° 38′ 07.06″ N, 95°38′ 40.68″ W), Veracruz, Mexico. The marsupials were immediately taken to the laboratory to be sedated through premedication with xylazine hydrochloride (0.2 mg/kg), induction with ketamine (0.5 mg/kg), and euthanized with intraperitoneal overdose of sodium pentobarbital. All marsupial specimens were subjected to necropsy.

### Morphological Analyses

The majority of collected worms were rinsed in saline (NaCl 0.85%), relaxed in chloral hydrate, fixed in hot 70% ethanol, stored in 70% ethanol, and examined in temporary whole mounts after clearing in Amman’s lactophenol. Measurements are given in millimeters unless specified otherwise and presented individually for holotype, allotype, and 2 paratypes (adult male and juvenile female). Line drawing was made using a drawing tube mounted on a stereoscope. Type specimens were deposited in the Colección Nacional de Helmintos (CNHE) Instituto de Biología (IB), Universidad Nacional Autónoma de México (IBUNAM), Mexico City, Mexico. For comparative purposes, the following *Gnathostoma* species from the United Sate National Museum (USNM), Smithsonian Institution, Washington, D.C, USA were examined: *G. oligomucronatum nomen nudum* (accession numbers: 88146, 88147 and 88148 deposited by Javier Almeyda Artigas); and *G. turgidum* from *D. virginiana* from CNHE, accession numbers: 4736, 4261, 4740, 4261.

One specimen (female), used for scanning electronic microscopy (SEM) studies, was processed according to [15] i.e., the specimen was fixed in 5% alkaline paraformaldehyde, and postfixed in osmium tetroxide; subsequently dehydrated with graded alcohols to acetone, dried at critical point (CO_2_), coated with a mix of gold–palladium, mounted on a metal stub and examined using a Hitachi SU1510 (Hitachi, Tokyo, Japan) scanning electron microscope at 10 kV at the Laboratorio Nacional de Biodiversidad (LANABIO), IB-UNAM. Ecological parameters follow [16].

### Molecular and Phylogenetic Analysis

Specimens for molecular analysis were preserved in 100% ethanol. DNA extraction was performed using the DNeasy Blood & Tissue (QUIAGEN) kit following the manufacturer’s protocol. A fragment of internal spacers transcribed ITS-1, complete 5.8S rDNA gene and a fragment of ITS-2 (hereinafter referred to as ITS for practical purposes), with the ITS4/ITS5 primers [17], with the following thermal profile: 1 cycle of 94 °C for 5 min; 35 cycles of 94 °C for 1 min, 52 °C for 1 min and 72 °C for 1 min; final elongation at 72 °C for 10 min. We also amplified partial Cytochrome C oxidase (*cox*1) and partial sequence of large ribosomal subunit 28S rDNA, with the following primers LCO/HCO [18] and 28SA/28SBout [19, 20], respectively, with the same thermal profile used for ITS, only interchanging to 48 °C annealing temperature for *cox*1 amplification.

PCR products were visualized by electrophoresis on agarose gel. Successful amplifications were purified using CentriSep 96 filter plates (ThermoFisher Scientific, Pittsburgh, Pennsylvania) with Sephadex G-50 (Cytiva, Marlborough, Massachusetts). Sequencing reactions included 0.4 µl BigDye Terminator v. 3.1 (Applied Biosystems, Waltham, Massachusetts), 2 µl Buffer 5x, 4 µl ddH2O, 1 µl of primer at 10 µM, and 3 µl purified PCR product (total volume 10 µl. Samples were purified using Sephadex G-50, then 25 µl de EDTA 0.5 mM was added to each sample and finally sequenced in an ABI-PRISM 3100 (Applied Biosystems® Waltham, Massachusetts) LANABIO, IB-UNAM.

The sequences generated here were aligned in MAFFT online version [21] with the sequences available in GenBank for *cox*1 and ITS independently. *Spiroxys ankarafantsika*, a member of Gnathostomatidae, was selected as outgroup. Phylogenetic relationships were constructed based on ITS sequences (644 bp and 26 terminals) as well as based on a *cox*1 matrix (1097 bp and 13 terminals); for more details of sequences used see Table 1. For the 28S rDNA region, a Blast search did not return any matches with *Gnathostoma* spp. due to the scarcity of available sequences for this genus in databases. Furthermore, the few sequences are notably short (e.g., *G. spinigerum* with 190 bp; acc. no. MT151891) making a reliable phylogenetic inference based on this region unfeasible. The inference criteria used was Maximum likelihood performed in IQtree [22] establishing 1000 pseudo replicates of ultrafast Bootstrap for nodal support value [23] and estimating automatically the substitution model that best fit to the data [24]. The best fit model for *cox*1 was TIM3 + F + I and for ITS was HKY + F + G4. Genetic distances were calculated with Mesquite [25] using the Kimura 2-parameters model according to [26].

**Table 1:** Metadata associated to taxa used in phylogenetic analysis of *Gnathostoma* species

Taxon names	Localities	Adult form	Larva	*Cox*1 GenBank acc no	ITS GenBank acc no	References
*Gnathostoma binucleatum*	Mexico		Unidentified fish	NC_080314		Díaz-Camacho et al. Unpublished
	Nayarit, Mexico	*Homo sapiens*			AY061740,	[27]
	Colima, Mexico		*Sciades guatemalensis*		FJ497054	[28]
	Sinaloa, Mexico		*Dormitator latifrons*		EU915243	[29]
	Guayaquil, Ecuador		*Rhamdia cinerascens*	AB180103	AB181159	[30]
*Gnathostoma doloresi*	Japan	*Sus scrofa*		AB180100	AB181156	[30]
			Unidentified eel		LC848949	[31]
*Gnathostoma miyazakii*	Canada	*Lontra canadensis*			FJ497055	[28]
*Gnathostoma hispidum*			Unidentified eel	JQ824056	JQ824057	Lee et al. Unpublished
	China	*Sus scrofa*		AB180102	AB181158	[30]
*Gnathostoma lamothei*	Veracruz, Mexico	*Procyon lotor hernandezii*			AY818004	[3]
	Tabasco, Mexico		*Gobiomorus dormitor*		EU334736	[32]
	EUA		*Monopterus* sp.		KF648543	[33]
*Gnathostoma mexicanum* n. sp.	“El Jobo” lagoon margins, Veracruz, Mexico	*Philander vossi*		PV761054	PV762167	This Work
		*Philander vossi*			OR428675	[34]
*Gnathostoma nipponicum*	Heilongjiang, China,		Unidentified fish		JN408313	Lee et al. Unpublished
	Japan	*Mustela* sp.		NC_034239		Sun et al. Unpublished
	Mie, Japan	*Mustela sibirica itatsi*		AB180101	AB181157	[30]
*G. spinigerum*	USA		*Monopterus* sp.		KF648553	[33]
	Cambodia		*Monopterus albus*	MK033970		[35]
	Nakhon Nayok, Thailand		*Monopterus albus*		AB181155	[30]
*Gnathostoma* sp.	Indonesia		Unidentified eel	JQ824052	JQ824055	Lee et al. Unpublished
*Gnathostoma* sp. II	Tlacotalpan, Veracruz, Mexico	*Philander vossi*			Z97173	[8]
*Gnathostoma turgidum*	USA		*Monopterus* sp.		KF648544, KF648546, KF648548	[33]
	Brazil	*Didelphis aurita*		KT894798		[36]
*Gnathostoma* sp. 1	Oaxaca, México		*Dormitator latifrons*	PQ141296		[34]
*Spiroxys ankarafantsika*	South Africa	*Pelusios castanoides*		MW545829	MW550280	[37]

## Results

**Gnathostomatidae** Railliet, 1895.

**Gnathostomatinae** Railliet, 1895.

***Gnathostoma*** Owen, 1836.

**Fig. 1:**
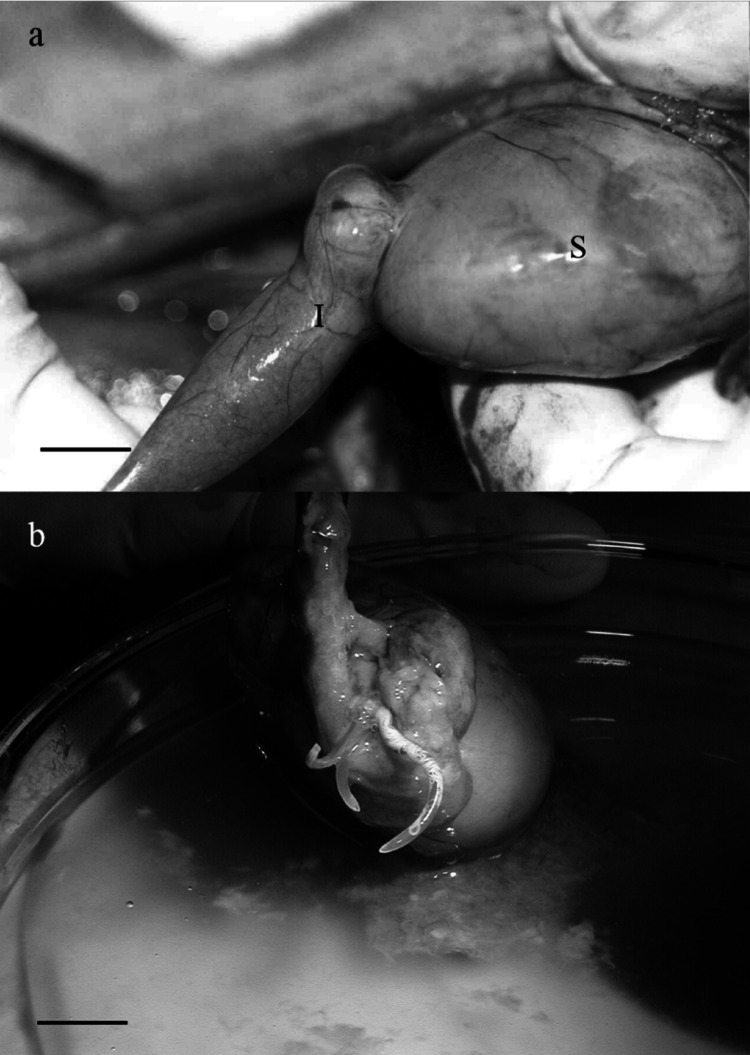
Infection site of *Gnathostoma mexicanum* n. sp. found in *Philander vossi*. **a** Tumor between stomach (S) and intestine (I), **b** Adult worms in the tumor. Scale bars, a = 20 mm, b = 5 mm

**Fig. 2:**
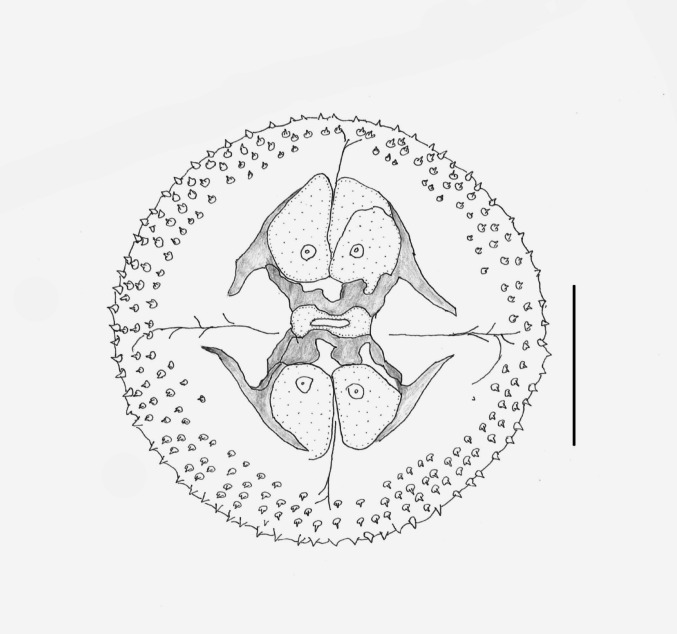
Apical view of the cephalic bulb of female of *Gnathostoma mexicanum* n. sp. collected in *Philander vossi*. Scale bar, 200 µm

**Fig. 3:**
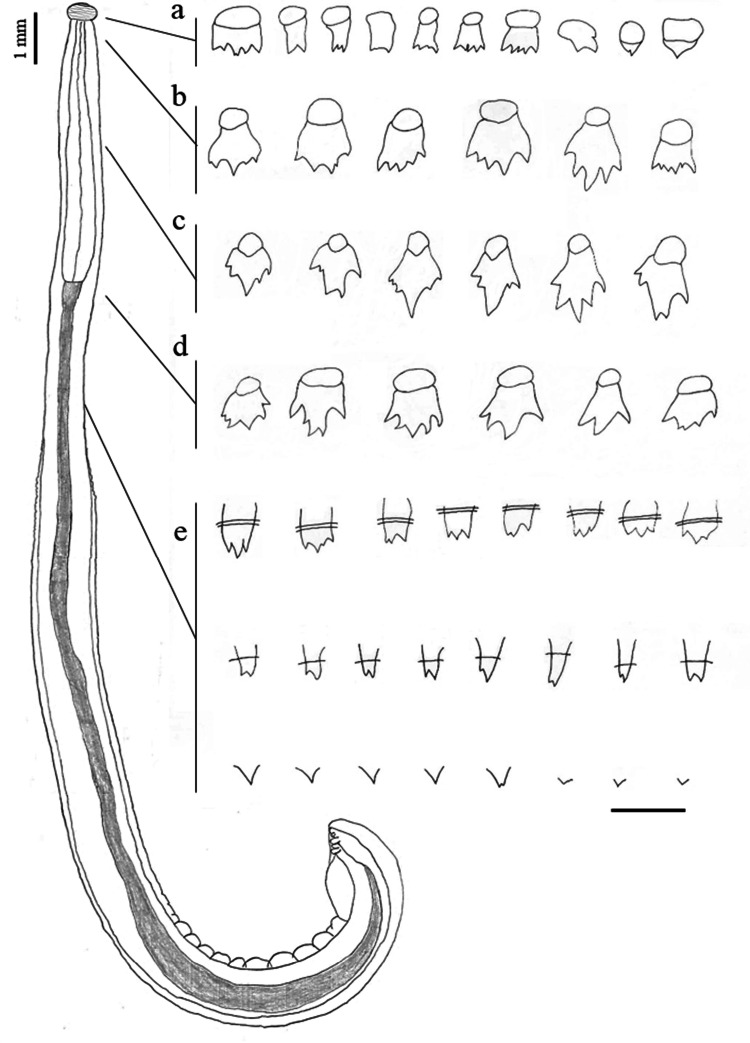
Morphology of body spines of male of *Gnathostoma mexicanum* n. sp. collected in *Philander vossi*. **a** first rows of body, **b** at level of the cervical papillae, **c** middle region of the esophagus, **d** esophagus-intestine junction, **e** subsequent levels of the spinated surface. Scale bar, 50 µm

**Fig. 4:**
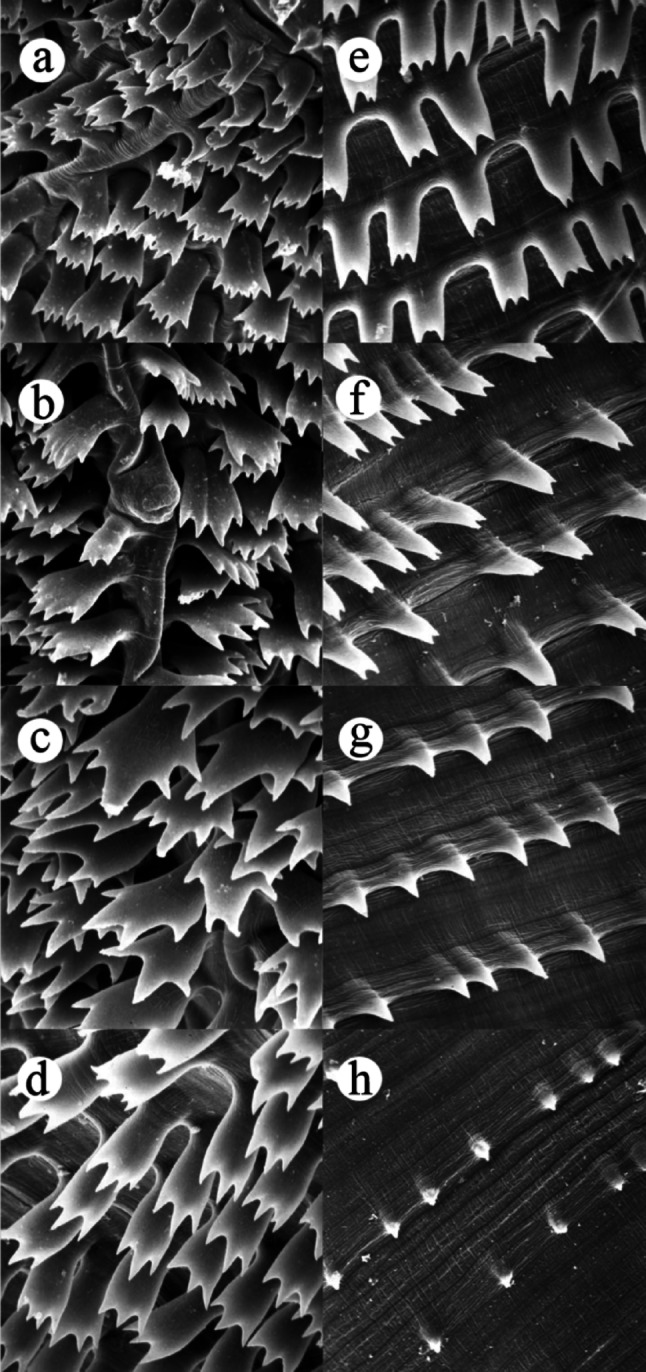
Scanning electron microscopy of adult (male) of *Gnathostoma mexicanum* n. sp. collected in *Philander vossi* **a** at neck level, **b** at cervical papilla level, **c** at the mid-oesophageal level, **d** at oesophagus-intestinal junction level, **e**–**h** subsequent levels of the spine surface

**Fig. 5:**
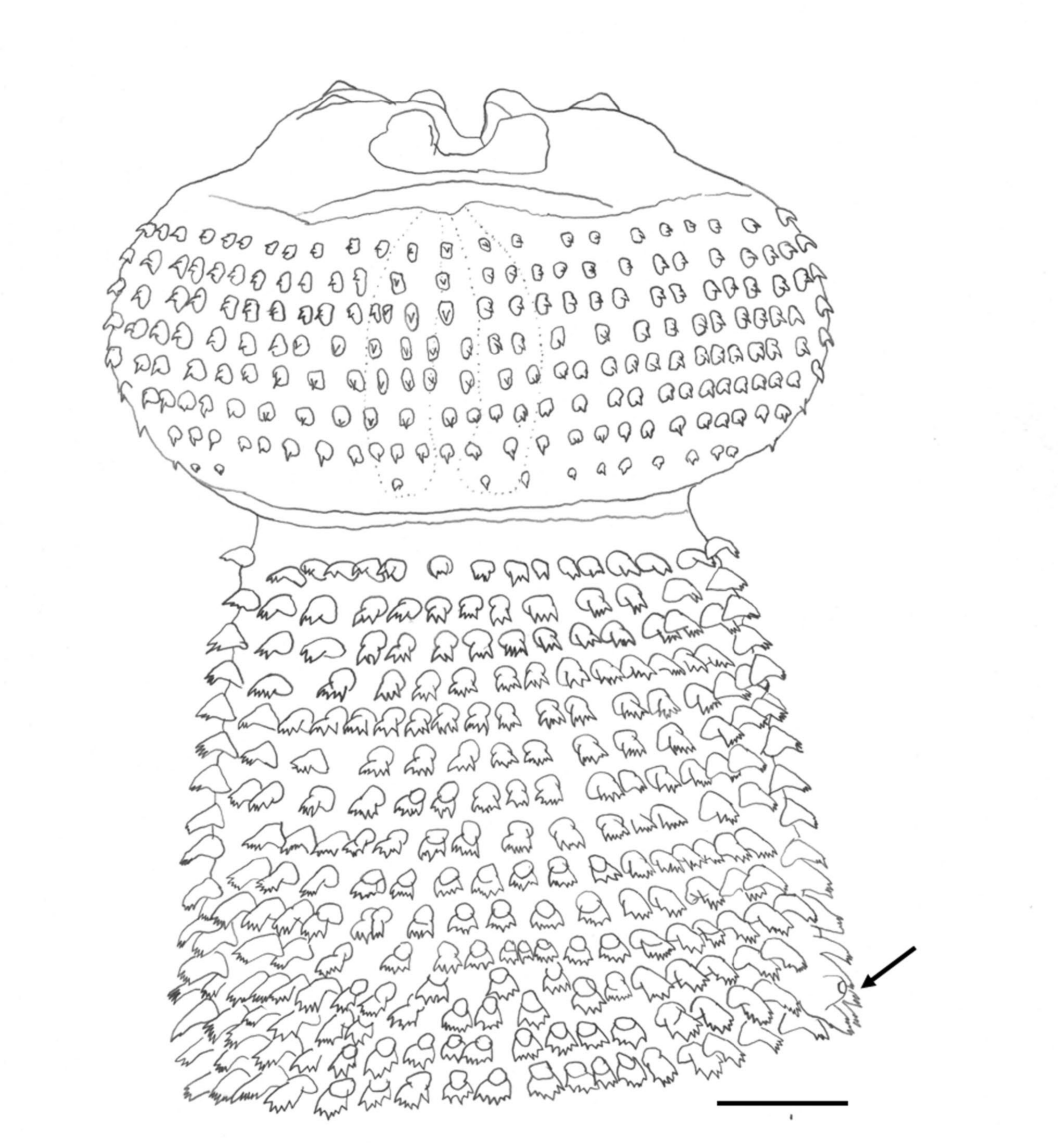
Anterior end of the male *Gnathostoma mexicanum* n. sp., collected from *Philander vossi*, showing arrangement of cuticular spines in cephalic bulb up to level of the cervical papilla (arrow). Scale bar = 100 µm

**Fig. 6:**
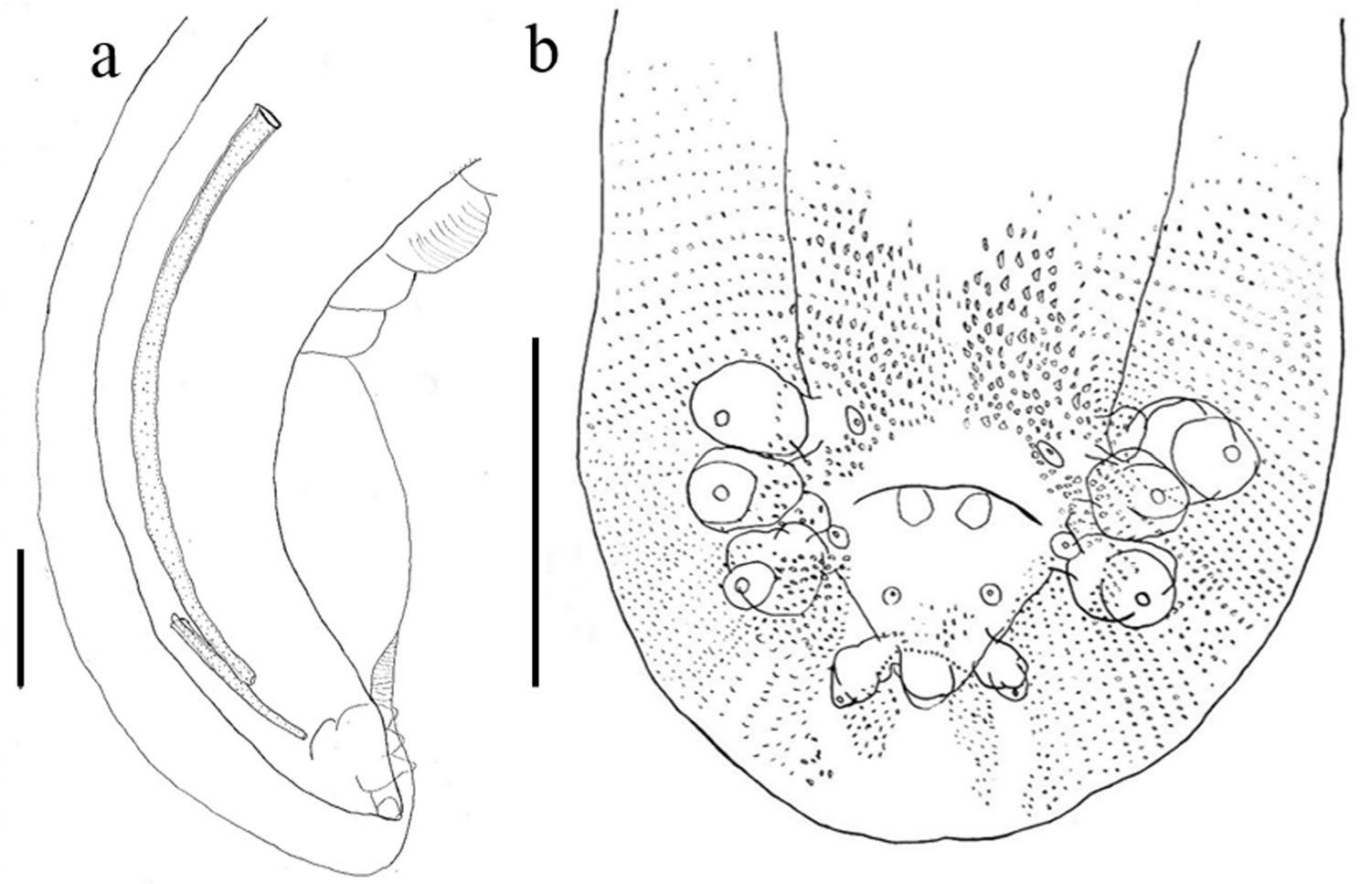
Posterior end of male of *Gnathostoma mexicanum* n. sp., parasite of *Philander vossi*. **a** Right lateral view and spicules, **b** Caudal bursa spination and genital papillae pattern. Scale bars = 100 µm

**Fig. 7:**
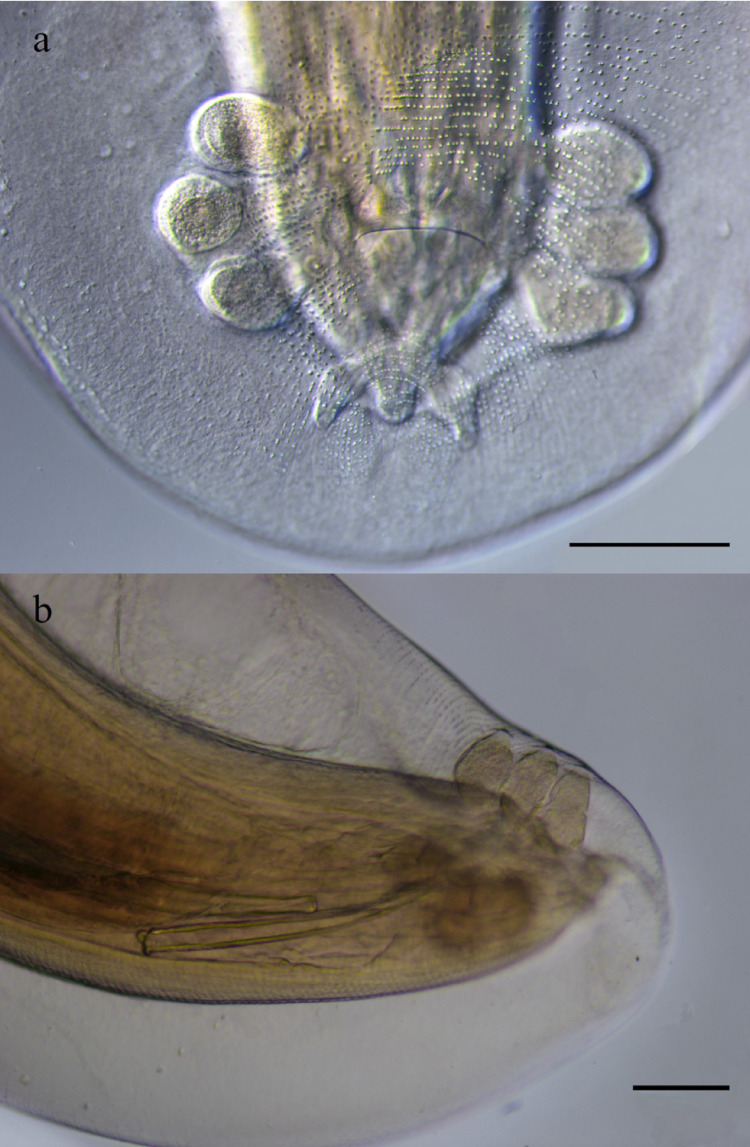
Caudal bursa of *Gnathostoma mexicanum* n. sp. parasite of *Philander vossi*. a Ventral view, **b** Right side view. Scale bar = 200 µm

***Gnathostoma mexicanum* n. sp.** (Figs. 1, 2, 3, 4, 5, 6 and 7).

### Taxonomic Summary

*Type host*: *Philander vossi*, Northern four-eyed opossum (Didelphimorphia: Didelphidae: Didelphinae).

*Site of infection*: in a single pyloric tumor, in which only the anterior third is buried; body in the lumen of the small intestine (Fig. 1).

*Type locality*: “El Jobo” lagoon margins (18° 37′ 58″ N, 95° 39′ 29″ W), proximities of Tlacotalpan City, Veracruz State, Mexico.

*Specimens deposited*: Holotype male CNHE: 12,849, allotype female CNHE: 12,850, Paratype male USNPC: 88,146, paratype female USNPC: 88,147, and paratype juvenile female CNHE: 12,287.

 Representative DNA sequences, GenBank accession numbers: *cox*1: PV761054, ITS: PV762167 and 28S *rDNA* PV762168.

*Etymology:* This species is named after the country from which the parasite was collected.

*Prevalence*: 50.0% (8 hosts collected, 4 infected).

*Mean intensity*: 0.85 (4 infected host/5 worms).

*ZooBank registration*: urn:lsid:zoobank.org:pub:83C98784-3567-4E81-AD89-2DC27570AE30.

Description based on 5 adult specimens, complete sets of measurements from the male holotype, the female allotype, 2 paratypes (male and female), and one juvenile paratype (female).

Shared structures somewhat larger in females than in males. Delicate and slender worms; anterior fourth of body slightly thinner than posterior three fourths. Cephalic bulb armed with 8–9 rows of uncinated hooklets and one pair of broad protruded lips with four small, submedian, double papillae, and lateral amphids not seen (Fig. 2). Ballonets 4 in number. Esophagus divided into short, anterior, muscular part and long, posterior, glandular part. The anterior half of body covered with transverse rows of cuticular spines of several shapes and different number of points (Figs. 3 and 4), as well as a pair of lateral cervical papillae between rows 13–16 (Fig. 5).

*Holotype*: total length 27.99; body width at anterior fourth 0.740; maximum body width at posterior half 1.170. Esophagus length 5.190 (18.540% respect to the total length of body), maximum width 0.510; four cervical sacs, 1.480 length (28.5%, respect to the total length of esophagus). Two cervical papillae between the transverse rows of cuticular spines, 14 (right) and 15 (left); excretory pore in row 33. Cephalic bulb 0.240 length by 0.540 width armed with eight complete transverse rows of hooks. Cephalic hooks 12–20 μm (15 ± 0.3, n = 10) long, 7–12 μm (9 ± 2, n = 10) wide at base; hooks of the last row smaller 5–7 μm (6 ± 1, n = 10) long, 5–5 μm (5 ± 0, n = 10) wide at base. Lips 29 μm long by 74 μm wide.

*Body spines arrangement as follows*: first rows posterior to neck multidentate (2 to 4 teeth, commonly 3) [lateral teeth thicker and slightly longer than central, 10–27 μm (19 ± 5, n = 10) long by 10–17 μm (13 ± 3, n = 10) wide in the region of the lateral teeth]; spines at cervical papillae level (4 to 6 points, often 5,) central teeth slightly longer than lateral, 29–39 μm (35 ± 3, n = 10) long by 17–27 μm (23 ± 4, n = 10) wide at region of the lateral teeth; large spines at mid-esophageal region, multidentate (4 to 6 teeth, generally 5; central tooth longer and thicker, occasionally forked), 54–61 μm (58 ± 2, n = 10) long by 39–49 μm (46 ± 3, n = 10) wide at lateral teeth level (Figs. 3 and 4). Spinous area extension 12.515 (44.70%). At the esophagous-intestinal junction, multi-tipped cuticular teeth (3–5 teeth, commonly 5) [if 3, the central teeth are longer and thicker than the lateral ones; frequently central teeth with 1 or 2 lateral teeth, on each side; some spines with the first pair of teeth slightly shorter than the 3 central ones; 61–69 μm (66 ± 3, n = 10) long, 29–42 μm (34 ± 4, n = 10) wide at the level of the lateral teeth]; the remaining spinous surface with the following subdivisions: (a) at the end of first third, with 2 and 3 equal teeth, sometimes central shorter; (b) at the middle of the second third, with 2 teeth of equal/unequal size and, (c) at the end of the spinous surface with single-tooth spine (Figs. 3 and 4).

Posterior half of body smooth, except in anal region, where transverse rows of single-tooth spine directed forward are observed. Caudal extremity with broad alae (Fig. 6) and eight pairs of caudal papillae, arranged as follows: four pairs of large, pedunculate, lateral papillae, one pair (1) precloacal, one pair (2) adcloacal and two (3, 4) postcloacal; the posterior pair much shorter than the others; the first three pairs lying close together, the fourth markedly separated from them, very close to the tip (Figs. 6 and 7). Four pairs of small, sessile, ventral papillae: one pair (A) preacloacal, one pair (B) adcloacal, and two (C, D) postcloacal (Figs. 3 and 6B). Tail 0.27 in length. Spicules delicate, unequal, with rounded apices; right spicule 0.64, much shorter than left (2.29); ratio between spicules 1.00:3.58 (Figs. 6 and 7).

*Allotype*: Total length: 36.29; body width: 8.4 in the forequarters; maximum width in the posterior half: 14. Esophagus length: 42 (11.61% of the total length) by 0.60 maximum width. Four cervical sacs, 1.52 (36.0%, respect to esophageal length). Cervical papillae between transverse rows 15 (rigth) and 16 (left) of cuticular spines. Excretory pore in row 37. Cephalic bulb 0.29 length by 0.55 width armed with nine complete transverse rows of hooklets. Body spines similar to those in male in the number of teeth, but with greater density in all regions. Extension of spinous area 47.60%. Vulva slightly postequatorial at 20.29 from anterior end (55.92%). Tail 0.21 in length. Embryonated eggs colourless. Eggshell with fine pits on entire surface and provided with cap-like thickenings in both poles. Eggs 62–70 μm (67 ± 5, n = 30) × 35–40 μm (38 ± 5, n = 30).

*Paratype (male)*: total length 23.04; body width at anterior fourth 0.71; maximum body width at posterior half 1.10; cervical papillae between transverse rows 14 and 15 of cuticular scales; cephalic bulb 0.29 length, 0.48 width, armed with eight complete and one incomplete transverse rows of hooklets; esophagus length 2.69, with a maximum width of 0.47; extension of spined area 55.08% of body length. Body spines arrangement similar in the number of teeth to that in holotype, but with lower density in all regions respect the allotype. Posterior half of body smooth, except in anal region, where transverse rows of one-tooth spines directed anteriorly are present. Caudal extremity with broad alae and eight pairs of caudal papillae, arranged as follows: four pairs of large, pedunculate, lateral papillae; one pair (1) precloacal, one pair (2) adcloacal and two (3, 4) postcloacal; the posterior pair much shorter than the others; the first three pairs are very close to each other, the fourth markedly separated from them, very close to the tip. Four pairs of small, sessile, ventral papillae: one pair (A) precloacal, one pair (B) adcloacal, and two (C, D) postcloacal. Tail 0.28 in length. Spicules delicate, uneven, with blunt apices; right 0.64, shorter than left (2.20); ratio 1.00:3.44.

*Paratype (female)*: Total length 38.84; body width at anterior fourth 0.97; maximum width at posterior half 1.71. Cervical papillae between transverse rows 14 and 15 of cuticular scales. Cephalic bulb length 0.35 and 0.61 width, armed with eight complete and one incomplete transverse row of hooklets; spinous area occupies 49.51% of the body length. Body spines arrangement similar to those in allotype, with an apparently higher density respect the holotype. Vulva postequatorial, situated at 20.38 from anterior end (52.47%); fertilized uterine eggs colorless, ovum unsegmented. Eggshell with fine granulations on entire surface, provided with cap-like thickenings in both poles. Eggs 66.3 μm (60.4–70.0, n = 10) × 36.8 μm (34.0–40.0, n = 10).

*Juvenil (female)*: Body length: 15.93 by 0.57 maximum width. Two cervical papillae on rows 13 (left) and 16 (right). The spiny surface of the body covers 8.73 of its length, reaching the level of the postequatorial vulva (54.7% of body length). Cervical sacs: 1.58 length on average. Esophagus: 2.53 (15.8% of body length) by 0.36 width. Body width at the esophagus-intestinal junction: 0.47. Head bulb: 0.20 × 0.34 length and width, respectively, with eight complete rows and one incomplete row of single-pronged hooks. Tail length: 0.24. Body spines similar to those of the allotype in the number of teeth, but with lower density in all regions.

### Molecular and Phylogenetic Results

**Fig. 8:**
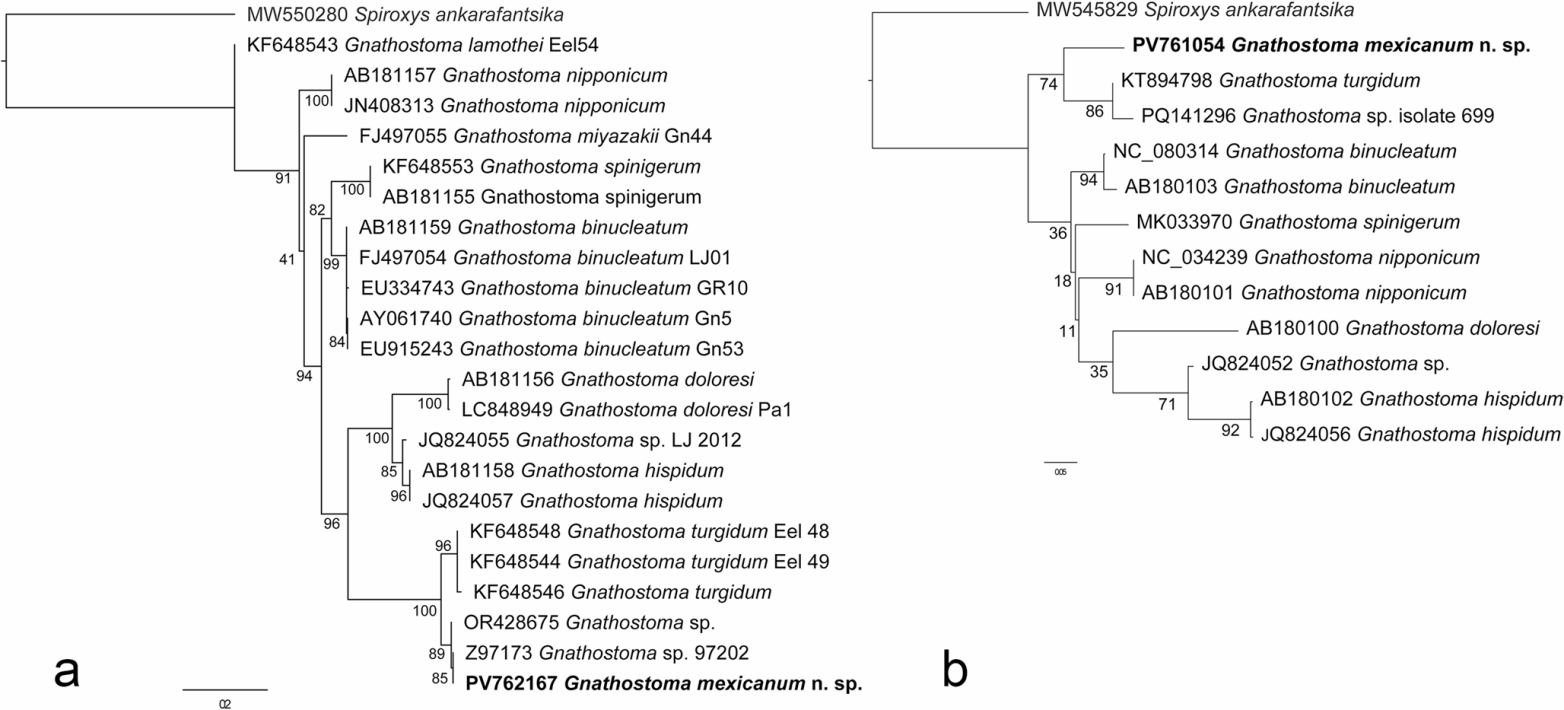
Maximum likelihood reconstruction of phylogenetic relationships between *Gnathostoma* species based on ITS (**a**) and *cox*1 (**b**) DNA sequences. The new species are denoted in bold

Both phylogenetic trees recover the new species as a sister taxon to *G. turgidum* with high support value (100%) in the ITS-based tree, but regular/low (74%) in the *cox*1-based tree. In general, internal relationships are not consistent between both trees, showing distinct topologies in early divergent branches (Fig. 8).

Comparative genetic divergences of ITS locus shows that the new species is nearly identical to unidentified larva *Gnathostoma* sp. II found parasitising *P. vossi* from Tlacotalpan, Veracruz (Table 2) and differs from the *G. turgidum* sequences by 6.4% on average, while intraspecific divergences do not exceed 0.5% in *G. turgidum* and 0.2% in the new species. Genetic divergence of *cox*1 between *G. mexicanum* and *G. turgidum* was 12.3% (Table 3).

**Table 2:** Pairwise genetic distances (K2P) of ITS region between *Gnathostoma* species

*Gnathostoma* species	(1)	(2)	(3)	(4)	(5)	(6)	(7)	(8)	(9)	(10)
(1) *G. turgidum*	0.5%									
(2) *Gnathostoma mexicanum* n. sp.	6.4%	0.2%								
(3) *Gnathostoma* sp. II	7.4%	0.4%	NA							
(4) *G. hispidum*	30.6%	31.1%	51.5%	1.7%						
(5) *G. doloresi*	36.5%	37.4%	57.8%	16.3%	0.6%					
(6) *G. binucleatum*	32.6%	32.6%	40.8%	23.1%	31.7%	0.1%				
(7) *G. spinigerum*	33.4%	33.8%	47.4%	24.7%	30.8%	12.9%	0%			
(8) *G. nipponicum*	32.6%	33.5%	45.4%	24.1%	30.7%	15.9%	19.4%	0%		
(9) *G. miyazakii*	38.1%	36.4%	45.3%	29%	35%	18%	23.6%	17.1%	NA	
(10) *G. lamothei*	28.3%	32.2%	43.6%	27%	34.3%	20.8%	24.1%	19.5%	19.7%	0%

**Table 3:** Pairwise genetic distances (K2P) of *cox*1 region between *Gnathostoma* species

*Gnathostoma* species	1	2	3	4	5	6	7	8
(1) *G. binucleatum*	1.8%							
(2) *G. nipponicum*	11.3%	0%						
(3) *G. spinigerum*	17.1%	13.4%	NA					
(4) *G. doloresi*	14.9%	13.8%	NA	NA				
(5) *G. hispidum*	12.3%	15.1%	NA	13.8%	0.5%			
(6) *Gnathostoma* sp.	19.0%	14.6%	NA	13.1%	7.5%	NA		
(7) *G. turgidum*	24.3%	15.5%	15.4%	NA	NA	NA	3.1%	
(8) *G. mexicanum* n. sp.	15.0%	15.3%	16.4%	NA	NA	NA	12.9%	NA

Remarks based on host specificity observed in the adult *Gnathostoma* spp., as well as the distribution, density and kind/shape of transversal rows of cuticular spines, the new species is readily distinguishable from the 13 valid species comprising the genus (Table 4). Comparison of the redescriptions by [5] of the American *Gnathostoma* species with both the morphological observations of our specimens and phylogenetic results indicates that the new species most closely resembles *G. turgidum*.

**Table 4:** Distinctive characters between species of the genus *Gnathostoma* and *Gnathostoma mexicanum* n. sp

Taxon	Character						References
	Type host	Country	Length (mm) (Male, Female)	Percentage of body surface covered by spines	Maximum number of teeth on the spines	Polar plugs in the egg	
*Gnathostoma spinigerum* Owen (1836)	*Panthera tigris*^1^	Englad	(12–31, 11–34)	Entire	4–5	1	[38, 39]
*Gnathostoma sociale* Leidy (1858)	*Mustela vison*^1^	USA	(24, 30–35)	34	5–6	1	[6, 40]
*Gnathostoma hispidum* Fedtschenko (1872)	*Sus scrofa ferus, S. s. domesticus*^1^	Hungary	(20, 26)	Entire	7–10	1	[38, 41]
*Gnathostoma turgidum* Stossich (1902)	*Didelphis azarae*^1^	Argentina	(40–42, 67–72), females up to 100 in length	50	5–10^*^	2	[5, 42, 43]
*Gnathostoma doloresi* Tubangui (1925)	*Sus scrofa domesticus*^1^	Philippines	(11–12, 30–41)	Entire	6–7	2	[38, 44]
*Gnathostoma americanum* Travassos (1925)	*Leopardus tigrinus*^1^	Brazil	?	75	5–7	2	[42]
*Gnathostoma nipponicum* Yamaguti (1941)	*Mustela sibirica itatsi*^2^	Japan	(20–28, 22–34)	50	4–7	1	[38, 45]
*Gnathostoma procyonis* Chandler (1942)	*Procyon lotor lotor*^1^	USA	(16–19, 20–26)	Entire	3–4	1	[46, 47]
*Gnathostoma miyazakii* Anderson (1964)	*Lontra cannadensis*^3^	Canada	(42, 41)	Bosses in the posterior half, only in males	6–7	1	[6]
*Gnathostoma malaysiae* Miyazaki and Dunn (1965)	*Rattus surifer*^1^	Tioman Island	(16, 22)	Entire	6	2	[48]
*Gnathostoma vietnamicum* Le-Van-Hoa (1965)	*Lutrogale perspicillata*^3^	Vietnam	(28, 43)	33	9	1	[49]
*Gnathostoma binucleatum* Almeyda-Artigas (1991)	*Leopardus pardalis*^1^	Mexico	(21–25, 15–27)	Entire	3^*^	1	[50]
*Gnathostoma lamothei* Bertoni-Ruiz, García-Prieto, Osorio-Sarabia and León-Règagnon (2005)	*Procyon l. hermandezii*^1^	Mexico	(13–19; 18–20)	Bosses in the posterior half	4–6^*^	1	[3]
*Gnathostoma mexicanum* n. sp.	*Philander vossi*^4^	Mexico	(23, 39)	45–48	4–6^*^	2	Present study

1 = Stomach, 2 = Esophagous, 3 = Kidney, 4 = Pylorus, *at level of cervical papillae

The main similarities are: (a) number of hooks from cephalic bulb; (b) spines present only at the anterior mid of body; (c) number and arrangement of caudal papillae; (d) position of the vulva, and (e) cap-like thickness at each end of the eggs. However, remarkable differences were also found: (1) fewer teeth on spines at level of cervical papillae (six vs. 10 teeth in *G. turgidum*); (2) spines from mid-esophagous level to intestine junction with maximum two lateral small teeth vs. maximum four lateral small teeth in *G. turgidum*; (3) minor body size (up to three times smaller than the Stossich’s species); (4) larger egg size (66.3 µm in the new species *vs*. 51.0 µm); (5) egg surface with fewer pits in *G. mexicanum* and (6) smaller cap-like length (3.48 µm vs. 4.35 µm in *G. turgidum*).

In addition, the new species produces tumors at the pyloric region of host with the anterior third of body embedded in the layers of the serosa. *Gnathostoma turgidum* forms individual tunnels penetrating the anterior part of body inside the mucus and muscularis of stomach, without specificity for some region. Larvae of four-stage and juvenils of *G. turgidum* have been reported infecting the liver of *P. opossum* [51, 52], which indicate that these larval stage nematode migrate through liver to reach the stomach where this mature and reproduce [53]. The finding of juvenile stages of *G. mexicanum* sp. n. exclusively at pyloric tumor, suggests that migration not encompass the liver. Notwithstanding, this require future investigations.

## Discussion

After the examination of the specimens labeled as *Gnathostoma oligomucronatum* (= *Gnathostoma* sp. II sensu [8]) by Bertoni-Ruiz et al. [5], a parasite of the pyloric region of *Philander vossi* (= *Philander opossum pallidum*) from El Jobo Lagoon, Tlacotalpan, Veracruz, Mexico, and deposited at USNM (88148, and 58535.02 by Javier Almeyda Artigas), the authors concluded that they correspond to *G. turgidum*. However, Almeyda-Artigas et al. [8] reported that ITS-2 rDNA sequences from this material support the distinction of *Gnathostoma* sp. II from *G. turgidum*. Unsurprisingly, the percentage of identity of the larva of *Gnathostoma* sp. II from Veracruz matches with *G. mexicanum.*

To our knowledge, no formal description of *G. oligomucronatum* has been published. Nevertheless, morphometric data from this material (USNM 88146, 88147, and 88148) align precisely with the diagnosis of the new species described herein. Consequently, *G. oligomucronatum* is relegated to *nomen nudum*.

With the species described here, there are now eight recognized *Gnathostoma* species in the Americas.

## Data Availability

No datasets were generated or analysed during the current study.
